# The Potential Important Role of Mitochondrial Rieske Iron–Sulfur Protein as a Novel Therapeutic Target for Pulmonary Hypertension in Chronic Obstructive Pulmonary Disease

**DOI:** 10.3390/biomedicines10050957

**Published:** 2022-04-21

**Authors:** Lillian Truong, Yun-Min Zheng, Yong-Xiao Wang

**Affiliations:** Department of Molecular and Cellular Physiology, Albany Medical College, Albany, NY 12208, USA; truongl@amc.edu (L.T.); zhengy@amc.edu (Y.-M.Z.)

**Keywords:** Rieske iron–sulfur protein, pulmonary hypertension, vasoremodeling, mitochondria, reactive oxygen species, DNA damage, inflammation

## Abstract

Chronic obstructive pulmonary disease (COPD) is the third leading cause of death worldwide, which is often due to pulmonary hypertension (PH). The underlying molecular mechanisms are poorly understood, and current medications are neither specific nor always effective. In this review, we highlight the recent findings on the roles of altered mitochondrial bioenergetics in PH in COPD. We also discuss the central role of mitochondrial reactive oxygen species (ROS) generation mediated by Rieske iron–sulfur protein (RISP) and review the contributions of RISP-dependent DNA damage and NF-κB-associated inflammatory signaling. Finally, the potential importance of mitochondrial RISP and its associated molecules as novel therapeutic targets for PH in COPD are meticulously discussed.

## 1. Introduction

Rieske iron–sulfur protein (RISP) in complex III of the mitochondrial electron transport chain is an essential molecule for reactive oxygen species (ROS) generation in pulmonary artery smooth muscle cells (PASMCs) [1,2,3,4]. This oxidative stress plays a significant role in the development of chronic obstructive pulmonary disease (COPD) and its associated pulmonary hypertension (PH). It has been shown that oxidative stress can mediate important cellular processes, such as PA vasoremodeling and vasoconstriction that lead to the development and progression of PH in COPD through the activation of various signaling pathways. In this review, we meticulously discuss the roles that RISP-mediated mitochondrial ROS play on DNA damage and inflammation on PA vasoremodeling, PA vasoconstriction, and PH in COPD. Understanding the functional importance of these complex, networking signaling pathways and their underlying mechanisms will shed light on helping to establish the potential specific, prime, and novel targets of RISP and associated signaling molecules for treating PH in COPD and other relevant diseases compared to the current non-specific medications.

### 1.1. Overview on PH in COPD

COPD is an umbrella term that encompasses chronic bronchitis and emphysema [5,6,7,8]. COPD is the third leading cause of the death worldwide. This chronic inflammatory respiratory disease is characterized by obstructed airflow caused by narrowing airways. Hyper-secretion of mucus (bronchitis), inflammation of the airway smooth muscle cells (ASMCs), and disruption of alveolar attachments (emphysema) contributes to narrowing airways seen in COPD [5,6,7,8]. Moreover, COPD can present in pulmonary and extra-pulmonary manifestations. Respiratory symptoms consist of frequent coughing and/or wheezing, mucus production, and shortness of breath.

Up to 91% of COPD patients will develop PH, characterized by an increase in pulmonary arterial pressure (P_PA_, >20 mmHg at rest) [9,10,11,12]. Prolonged PH may account for cardiac dysfunction, contributing to the high mortality rate of COPD patients. These COPD patients are at higher risk for cardiac arrhythmias, myocardial infarction, and congestive heart failure [13]. Approximately two-thirds of COPD patients display right ventricular hypertrophy, secondary to increases in P_PA_, as assessed by right heart catheterization. Thus, pulmonary manifestations of COPD (i.e., hypoxemia, inflammation, arterial remodeling) often cause and exacerbate cardiac manifestations [6,12], thereby worsening COPD symptoms, and increasing risk of death. Although PH in COPD is fairly common, the underlying mechanisms are still largely unknown.

### 1.2. Causes and Risk Factors

COPD often occurs due to chronic exposure to irritants, specifically in the lungs and airways. Cigarette smoke (CS) is recognized as an important risk factor in the development of COPD [5,7,14]. Exposure to CS or nicotine causes, exacerbates, and prolongs the symptoms and features of COPD and associated PH, where up to 90% of COPD cases can be attributed to cigarette or e-cigarette smoke [15,16,17,18]. Nicotine is the major active component of cigarettes, e-cigarettes, and nearly all other tobacco-derived products. More importantly, nicotine inhalation may replicate almost all major features of COPD [19,20,21,22] and also cause PH [23,24].

### 1.3. Treatments

Currently, there is no cure for COPD. Limiting exposure to COPD-exacerbating triggers and management of COPD symptoms are the primary approaches of slowing disease progression and improving quality of life [5,7,8]. The current optimal treatments utilize both non-pharmacological and pharmacological interventions. The former primarily include lifestyle changes such as smoking cessation and avoiding airborne irritants (i.e., secondhand smoke and other air pollutants). The latter often use various drugs depending on severity of symptoms, triggers, patient conditions, and comorbidities, to avoid complications and risk of death. Pharmacological treatments target: (1) increasing airflow and (2) reducing inflammation [5,7]. Airway dilators or bronchodilators are used to increase airflow, working as β2 agonists or muscarinic receptor antagonists. Anti-inflammatory agents target systemic inflammation associated with COPD in order to reduce exacerbations and disease progression. Thus, inhaled corticosteroids are often prescribed in combination with bronchodilators. However, corticosteroids also have several risks, including risk of pneumonia and withdrawal symptoms (steroid withdrawal syndrome) once treatment is completed or ceased.

Although current treatments for COPD primarily focus on symptom- and trigger-management to slow disease progression, there is still no cure for COPD. Furthermore, it is necessary to develop specific treatments that target the underlying mechanisms that lead to the development and progression of COPD and its associated PH.

## 2. Cigarette Smoking and Nicotine

As mentioned above, up to 90% of COPD cases can be attributed to CS [14,21]. Chronic exposure to CS and nicotine is detrimental for the cellular environment, including cell injury, infiltration of inflammatory cells in the lung, increase in ROS, oxidative stress, and pro-inflammatory cytokines. These cellular responses to cigarette smoke and nicotine call for investigation as they promote the development and progression of PH in COPD in both the airway and pulmonary vasculature.

### 2.1. Airway Cellular Responses

ASMCs are the primary type of cells responsible for airway remodeling, contributing to the narrowing airways in COPD [25,26,27]. Airway remodeling not only causes an increase in structural respiratory resistance to limit airflow, but also in functional respiratory resistance to further restrict airflow due to the increased number of ASMCs and subsequent airway hyperresponsiveness (AHR) in response to irritants and agonists [27,28,29,30]. AHR is a key feature in COPD and is reversible with bronchodilators. Thus, these types of drugs are the first-line medications for COPD.

Inflammation in the medial wall of the airway also plays a dominant role in airway remodeling. This inflammation may be primarily attributed to ASMCs in response to irritants such as CS or nicotine, as they are the predominant cell type found in the airway medial wall. It has been shown that pro-inflammatory cytokines such as interleukin (IL)-1β and tumor necrosis factor-β (TNF-β) are upregulated in the airways and, thus, cause ASMC proliferation, airway remodeling, AHR, and airflow restriction in COPD [25,28,29]. Interestingly, these cellular responses of ASMCs can be secondary to the increased ROS and/or DNA damage [31].

It is also noted that bronchoalveolar lavage fluid (BALF) collected from COPD patients show increased levels of matrix metalloproteases (MMPs), enzymes responsible for degrading the extracellular matrix [32,33,34]. The increased MMPs in COPD may be attributed to the increase in recruitment and infiltration of inflammatory cells including neutrophils and macrophages [32,33,34]. These cellular responses ultimately result in alveolar destruction and subsequent airway obstruction, contributing to COPD.

### 2.2. Pulmonary Arterial Cellular Responses

The airway remodeling, AHR, and airflow restriction in COPD may contribute to the cellular responses in PASMCs. It is clear that CS, nicotine inhalation, and other factors may directly cause cellular responses in PASMCs, leading to PA vasoconstriction, vasoremodeling, and PH [9,10,21].

PH in COPD is associated with the high mortality rate of COPD patients [10,35,36]. This devastating disease is primarily manifested by increased pulmonary vascular resistance (PVR), which occurs due to increased PA vasoconstriction and remodeling. These two major cellular responses are highly determined by hyperresponsiveness, increased proliferation, and decreased apoptosis of PASMCs. Hypoxic pulmonary vasoconstriction (HPV) is an adaptive mechanism in response to alveolar hypoxia (a decrease in oxygen) [37]. This mechanism is used to match perfusion to ventilation in smaller resistant pulmonary arteries. Vasoconstriction is highly controlled by Ca^2+^ signaling in PASMCs, specifically the release of Ca^2+^ from intracellular stores (sarcoplasmic reticulum, SR) and/or influx from extracellular compartments through various ion channels [3,38].

Vascular remodeling, or vasoremodeling, is characterized by changes to the vasculature. This process often involves hyperproliferation of key cells (i.e., PASMCs), basement membrane thickening, fibrosis, and deposition of collagen [39]. Where airway remodeling plays a significant role in COPD, vasoremodeling involving increased PASMC proliferation contributes to COPD-associated PH. Several studies have shown the significant role that cigarette or tobacco smoke plays on vasoremodeling. Interestingly, cigarette or tobacco smoke-induced ROS generation contributes to airway and pulmonary vascular remodeling in COPD [21,25]. A study by Zhu et al. has shown that tobacco smoke-induced ROS generation activates calpain, which is necessary for airway or PA remodeling [31]. Another study by Churg et al. has shown that cigarette smoke can induce activation of several growth factors and pro-collagen synthesis in the airways [25]. Again, these studies suggest the highly effective role of cigarette smoke and its major component nicotine in COPD and its associated PH.

## 3. Ca^2+^ Signaling

As the most common second messenger, Ca^2+^ signaling plays a significant role in many cellular responses, including PA vasoconstriction and PH in COPD. Events that lead to increases in intracellular Ca^2+^, such as hypoxia, can contribute to constriction and proliferation of SMCs [3,38,40,41]. This highly-regulated Ca^2+^ signaling can activate other signaling pathways to cause synergistic cellular responses in both normal and pathophysiology via Ca^2+^-induced ROS generation (CIRG) or Ca^2+^-induced Ca^2+^ release (CICR), as detailed below.

Many ion channels generate and regulate Ca^2+^ signaling through intracellular Ca^2+^ release or extracellular Ca^2+^ influx in PASMCs [42]. Intracellular Ca^2+^ stores, such as the SR, may release Ca^2+^ through ryanodine receptor (RyR) and/or inositol 1,4,5-trisphosphate receptor (IP_3_R) channels [3,43]. This release can initiate and promote subsequent Ca^2+^ signaling. RyRs, including all three subtypes (RyR1, RyR2, RyR3) have been shown to play a major role in hypoxia-induced cellular responses. However, studies have shown that RyR2 plays a dominant role [3]. A series of our studies have revealed that RyR2 is normally associated with and inhibited by FK506 binding protein 12.6 (FKBP12.6) in PASMCs, and roles of RyR2 in hypoxia-evoked cellular responses are strongly medicated by dissociation of RyR2 and FKBP12.6 complex [44,45,46].

On the SR of PASMCs, three subtypes of IP_3_Rs (IP_3_R1, IP_3_R2, and IP_3_R3) have been found. IP_3_R1 has been shown to play a significant role in mediating intracellular Ca^2+^ release, which is highly associated with the pathophysiology in PH. Further studies have unveiled that the role of IP_3_R1 in hypoxic Ca^2+^ release is a result of the increased IP_3_ production due to activation of phospholipase C (PLC, particularly PLCγ1) [43,47].

Several Ca^2+^ channels have been shown to regulate Ca^2+^ influx by altering membrane potential. L-type voltage-dependent Ca^2+^ channels (LTVDCCs) are responsible for membrane depolarization-dependent Ca^2+^ influx in PASMCs [42,48], whereas voltage-gated K^+^ (K_V_) channels are responsible for regulating membrane potential [49]. Inhibition of K_V_ channels can activate LTVDCCs due to the depolarization of the cell membrane. In PH in COPD, hypoxia can also inhibit K_V_ channels, causing Ca^2+^ influx and vasoconstriction. Increases in cytosolic Ca^2+^ can also influence cell proliferation and inhibit apoptosis, which may lead to the vascular remodeling seen in PH.

Transient receptor potential (TRP) channels are a family of non-selective cation channels. The upregulation of TRP channels have been associated with increased Ca^2+^ influx and implicated in PH development and progression. Canonical TRP (TRPC) channels are a subfamily of TRP channels that are expressed in pulmonary arteries. Seven isoforms, TRPC1 through TRPC7, are expressed; however, TRPC1 and TRPC6 have been shown to play a dominant role in Ca^2+^ release responses in PASMCs [44,50]. Specifically, cigarette smoke- and nicotine-induced PA vasoconstriction and remodeling is highly influenced by the upregulation of TRPC1 and TRPC6 [51]. Interestingly, a study by Hong et al. showed that CS and nicotine exposure lead to increased TRPC6 expression [52].

In addition to expression in the pulmonary arteries, studies have shown major involvement of TRPC6 in airway cellular responses induced by cigarette smoke and nicotine exposure. These studies indicate that nicotine causes increased proliferation of ASMCs, and that this nicotine-induced response relies on TRPC6-dependent Ca^2+^ influx through α-7 nicotinic acetylcholine receptor (α7nAChR) [52]. Treating human ASMCs with α7nAChR antagonists attenuates nicotine-induced proliferation; treatment of TRPC6 siRNA with or without the presence of nicotine inhibits cell proliferation. Given that increased intracellular Ca^2+^ levels promote cell proliferation and nicotine-induced increase in TRPC6 expression, Ca^2+^ influx through α7 nAChR may be responsible for the hyperproliferation of PASMCs leading to PA vasoremodeling and PH in COPD.

## 4. Reactive Oxygen Species

ROS are chemically reactive molecules that contain oxygen. The well-regulated ROS are very important to maintain normal cellular homeostasis, where unregulated ROS generation can lead to detrimental effects [53,54]. These effects include DNA damage, protein oxidation, lipid peroxidation, etc. The development and progression of asthma, COPD, PH, and other respiratory diseases can be highly attributed to oxidative stress. In PASMCs as well as other cell types, there are a number of sources for ROS generation. The role of ROS in PASMCs has been shown to be involved in PA vasoconstriction and remodeling leading to PH [2,3,4,44,55,56,57]. However, little is known about its precise signaling mechanisms; thus, more directed studies to address this important questions have reached a new level of urgency.

### 4.1. Sources of ROS

Mitochondria are the primary and most important source of ROS generation. Due to their oxygen-rich environment and involvement of the mitochondrial electron transport chain (ETC), electron reduction of oxygen to superoxide is common and thus a major reaction producing ROS. Studies have shown that mitochondria can also produce hydrogen peroxide (H_2_O_2_). Although there are four complexes in the ETC, complexes I and III are highly regarded as the main contributors of mitochondrial ROS generation [1,2,58,59,60]. Within these complexes, increased electron movement and potential electron leakage favor and contribute to ROS generation. In addition to electron movement within these complexes, a high NADH/NAD+ ratio in the mitochondrial matrix has also been shown to contribute to ROS generation [61,62].

Our laboratory and others have shown that RISP, a catalytic subunit of mitochondrial complex III, is a major contributor to ROS generation. Specifically, knockdown of RISP in PASMCs inhibits hypoxia-induced increase in mitochondrial ROS generation, while its overexpression promotes the hypoxic response [1,2,3,4,44].

Another source of ROS generation is well-studied nicotinamide dinucleotide phosphate (NADPH) oxidase (NOX) [63,64,65,66]. Regulation of NOX-mediated ROS generation is highly controlled through interacting with cytoplasmic and membrane-associated proteins, as well as responding to intracellular stimuli, such as Ca^2+^. One study has shown that phosphorylation and subsequent modulation of NOX activation may be regulated by protein kinase-C (PKC). Our laboratory has also shown that RISP-dependent mitochondrial ROS can activate PKCε and then NOX to induce further ROS generation, i.e., ROS-induced ROS generation (RIRG) [57,67,68]. Importantly, these studies support that mitochondrial ROS generation precedes non-mitochondrial ROS generators, such as NOX [69]. This RIRG provides a positive feedback mechanism that may further increase hypoxia-evoked cellular responses [67]. Enzymes including xanthine oxidase, dual oxidases, cyclooxygenases, and lipoxygenases may act as other sources of ROS generation and, thus, play a significant role in hypoxic responses [53,70,71].

### 4.2. Regulatory Mechanisms of ROS Signaling

Unregulated ROS generation plays a large role in many cellular processes and can lead to disease development. ROS can influence different signaling pathways through their interaction with key molecules or their residues. These aberrant interactions can lead to unregulated and premature activation of signaling pathways associated with the development and progression of devastating diseases.

Mitogen-activated protein kinase (MAPK) cascade signaling is involved in numerous cellular processes including cell growth, differentiation, survival, and death [54,72]. ROS have been shown to activate MAPK downstream JNK and ERK signaling pathways. One study indicates that PA vasoremodeling and PH are dependent on ROS-mediated ERK activation and subsequent phosphorylation [54]. This ERK signaling activation can influence collagen synthesis and proliferation in both ASMCs and PASMCs, where collagen synthesis and cell proliferation both are key characteristics of remodeling [31]. NOX4-derived ROS generation, secondary to TGF-β stimulation, has shown to mediate PA vasoremodeling, contributing to COPD-associated PH development [73]. Additionally, H_2_O_2_ can induce the phosphorylation of PLC, thus producing diacylglycerol (DAG) and IP_3_, the ligand involved for IP_3_R-mediated Ca^2+^ signaling [54]. As previously mentioned, mitochondrial ROS-induced activation of PKCε can cause activation of NOX to induce further ROS generation, (i.e., RIRG) [57].

There are several signaling pathways that maintain cellular redox and mediate responses to oxidative stress, including Kelch-like ECH-associating protein 1 (Keap1)-nuclear respiratory factor (Nrf)-antioxidant response element (ARE) signaling. Increased ROS generation can disrupt Keap1-Nrf-ARE-dependent signaling by causing dissociation of regulatory inhibitor Keap1 from Nrf [54]. This is primarily mediated by the oxidation of specific cysteine residues or activation of kinases such as PKC, MAPK, or PI3K that phosphorylate Nrf, which are highly regulated by ROS.

### 4.3. The Interplay of Ca^2+^ and ROS Signaling

Studies suggest that the interaction between ROS and Ca^2+^ signaling may finely regulate cellular responses and other signaling pathways. Given that these signaling pathways have overlapping importance, disruption, or dysfunction to either signaling pathways may affect the other and contribute to pathophysiology in disorders such as PH in COPD [74,75,76].

Hypoxia, a key initiator in the development of PH in COPD, can lead to an array of downstream cellular responses in PASMCs. Studies have shown that the mitochondria can act as an oxygen sensor [58,59,77,78]. HPV in PH may cause hypoxia-induced change in ROS that can inhibit K^+^ channels. This inhibition of K^+^ channels in turn causes membrane depolarization and cell contraction due to an increase in cytosolic Ca^2+^ [79]. Sustained hypoxia can activate Rho kinase, leading to Ca^2+^ sensitization, thus reinforcing PA vasoconstriction and PH [80,81,82].

Additionally, ROS-induced Ca^2+^ signaling through oxidation of key residues on several ion channels to increase Ca^2+^ influx. It has also been shown that intracellular Ca^2+^ can mediate ROS generation, causing an interactive role between Ca^2+^ and ROS signaling. Besides the mitochondria, Ca^2+^ can regulate several enzymes that generate ROS, including NOX and nitric oxide synthase (NOS) [83,84]. Antioxidant enzymes, such as catalase and GSH reductase, can be activated by Ca^2+^. It has also been shown that Ca^2+^ can increase expression levels of superoxide dismutase (SOD), an enzyme involved in superoxide catalysis to H_2_O_2_ [85]. These Ca^2+^-mediated responses are also known as CIRG, or Ca^2+^-induced ROS generation. It is also well known that ROS can stimulate Ca^2+^ release from intracellular Ca^2+^ stores, termed ROS-induced Ca^2+^ release (RICR). Evidently, all these processes play an important role in the development of PH.

A recent study has shown that ROS can directly influence RyR-mediated Ca^2+^ release through interaction with important cysteine 3602 (Cys3602) in HEK293 cells. It has been reported that Cys3602 is important for the function of RyR2 and RyR2-mediated SR Ca^2+^ release in HEK293 cells, evident by the findings that application of the thiol alkylating agent N-ethylmaleimide induces Ca^2+^ release in cells expressing wild type RyR2, but not in cells expressing a mutant RyR2, where Cys3602 is mutated to alanine [86]. This study indicates that this cysteine residue is highly important and is a major site for mediating the effect of thiol alkylating agents on RyR2. Additionally, some TRP channels, including TRPC3, are highly regulated by ROS [87,88]. Taken together, ROS may regulate Ca^2+^-mediated cellular responses in PASMCs, including contraction, proliferation, and apoptosis, all of which are associated with the development and progression of COPD and its associated PH.

#### The Role of RISP on Ca^2+^ Regulation

Ca^2+^ signaling in PASMCs play an important role in PH. ROS-regulated Ca^2+^ signaling through processes, such as RICR, contributes to this disease development. Our laboratory recently demonstrated that RISP knockdown blocks hypoxia-induced RyR2 oxidation and subsequent PH [44]. Intravenous injection of lentiviral RISP shRNAs silenced RISP expression in smooth muscle-specific tissues (i.e., PAs). Importantly, RISP KD significantly decreased RyR2 activity in PASMCs from hypoxic mice. RyR2 oxidation was assessed by immunoprecipitation of RyR2 protein bound by carbonyls, followed by immunoblotting of 2,4-dinitrophenyl (DNP). Increased RyR2 oxidation was detected in PASMCs in hypoxic mice, whereas RyR2 oxidation was blocked by RISP KD [44]. This study suggests that RISP plays a critical role in regulating RyR2-mediated Ca^2+^ signaling.

HPV, initiated by hypoxia, can increase P_PA_ and lead to PH. This innate mechanism of vasoconstriction is highly regulated by Ca^2+^. However, studies have also shown that RISP-mediated ROS signaling may act to regulate HPV [2,3,4,60,76]. Using a RISP KO animal model, lung slices from RISP KO mice demonstrated abolished hypoxia-induced increases in Ca^2+^ signaling of the PA. Additionally, in vivo RISP depletion attenuated hypoxia-induced increase in RVSP. Thus, these studies suggest RISP-generated ROS can mediate HPV, an important factor in the development of PH.

### 4.4. The Interplay of ROS, Nicotine, and Smoking

As mentioned earlier, CS and nicotine have been shown to enhance ROS generation in PASMCs. High concentrations of free radicals in cigarette smoke may directly contribute to the increased cytosolic ROS levels in PASMCs [89,90,91]. More importantly, CS and nicotine can activate intracellular ROS generation systems and lead to large intracellular ROS production [90,91,92,93]. With many COPD patients being chronic smokers, the constant exposure to CS can lead to oxidative stress. Thus, understanding the cellular effects of increased ROS generation in COPD patients can help to guide future research into more targeted therapeutic treatments.

### 4.5. The Role of ROS in PH in COPD

Studies have shown that both COPD-associated PA vasoremodeling and vasoconstriction can be attributed to aberrant ROS generation [94]. Increased ROS generation secondary to CS or nicotine inhalation can exacerbate inflammation in COPD patients, as shown by injury to bronchiolar epithelial cells, infiltration of neutrophils and macrophages, and expression of oxidative stress markers and production of pro-inflammatory cytokines [7,94]. Analysis of sputum and BALF samples from COPD patients illustrate increased nuclear factor (NF)-κB activity. Interestingly, NOX4-derived ROS have been shown to activate NF-κB and MAPK signaling [95,96,97,98].

Complementary to a pro-inflammatory environment, COPD patients display reduced anti-inflammatory defense, as illustrated by decreased expression of glutathione (GSH), a master antioxidant, in sputum or BALF collected from COPD patients [99]. In addition to reduced GSH expression, COPD patients also display decreased SOD, reduced catalase activity, and reduced GSH peroxidase (GPx) levels [89,99,100]. Collectively, reduced anti-inflammatory defense coupled with a pro-inflammatory environment in response to increased ROS may contribute to the observed oxidative stress in cigarette smoking-related COPD.

Additionally, mitochondrial dysfunction is linked to an increase in ROS generation in COPD patients compared with non-smoking healthy human subjects [101]. Mitochondrial damage, which can initiate from increased H_2_O_2_ and/or the presence of CS, display decreased membrane potential, impaired oxidative phosphorylation, and ATP production, as well as aberrant Ca^2+^ signaling [101,102]. It has also been shown that oxidative stress-induced mitochondrial dysfunction may drive COPD-associated inflammation.

### 4.6. Therapeutic Effect of Antioxidants in PH in COPD

Considering the central role of ROS in the development of PH in COPD, we must consider the potential role for antioxidants as therapy [103]. In animal studies, several compounds with antioxidant properties have been used to block the progression of PH in COPD. These compounds consist of pyrrolidine dithiocarbamate (PDTC), SOD, allopurinol, sulfur dioxide and more [104,105]. However, it is important to note that cellular homeostasis requires a balance between ROS production and scavenging. Use of general antioxidants may affect the complex balance that either promote homeostatic redox signaling or drive harmful oxidative stress. Thus, therapies that target specific molecules involved in ROS generation, e.g., RISP in mitochondrial complex III, may be more effective and specific in treating PH in COPD.

## 5. DNA Damage

DNA is chemically unstable and vulnerable to hydrolysis, oxidation, and methylation. DNA damage, including single-stranded DNA breaks (SSBs) and double-stranded DNA breaks (DSBs), can occur from endogenous and exogenous genotoxic agents (such as ROS and reactive nitrogen species (RNS)) and byproducts of oxidative stress (aldehydes produced by lipid peroxidation) [106]. Exogenous agents include environmental factors, which include exposure to mutagenic chemicals or physical agents, such as UV or ionizing radiation. Exposure to cigarette smoke and its major component, nicotine, has been linked to DNA damage, often associated with the development of lung cancer [107]. Oxidative DNA damage is inevitable to chronic exposure to cigarette smoke or nicotine inhalation. Increased levels of superoxide or highly reactive hydroxyl radicals can react with DNA.

Increased DNA damage and dysfunctional DNA damage repair (DDR) signaling have been shown to be involved in PH pathogenesis [107,108,109]. These DDR pathways work in a multi-faceted approach, which includes restoring DNA double-strands activation of DNA damage checkpoint kinase 1 and (Chk1 and Chk2) to avoid furthering damaged DNA, transcriptional alteration, and apoptosis signaling in cases of irreparable damage.

### 5.1. Major DNA Damage and Repair Signaling Pathways

There are a number of signaling pathways to maintain genomic integrity, regulate cell cycle progression and apoptosis. These pathways detect DNA lesions (SSBs and DSBs) and signal their presence to promote their repair through biomolecule markers. Defective mechanisms can lead to decreased repair, transmission of mutated DNA, and development DNA damage-associated diseases such as cancer. The well-known two major signaling DNA damage response signaling pathways are ataxia telangiectasia-mutated (ATM) and ATM-related (ATR) [110,111].

Both ATM and ATR regulate cell cycle checkpoint pathways to induce cell cycle arrest and DNA repair to prevent transmission of damaged DNA. ATM and ATR respond to different types of DNA lesions. ATM primarily responds to DSBs [112], whereas ATR is activated by DSBs and protects replicating chromosomes [110,113]. Although ATM and ATR are similar in that they work to maintain cellular homeostasis, their pathways are distinct and complex. Many reviews have covered the extensive processes of ATM and ATR activation; thus, herein we will principally discuss the functional roles of oxidative stress and inflammation in their signaling pathways.

ATM activation is readily triggered by increased ROS generation. The activation of ATM induces a large span of signal transduction pathways that intersect DNA damage repair, metabolism, and pathways that include protein translation and transcription [110,112]. The MRN complex binds dsDNA ends acting as a sensor that promotes ATM recruitment. The interaction between ATM and MRN component NBS1 is also important for ATM recruitment and DNA binding. Chk2, a well-known effector of ATM signaling, is phosphorylated at threonine 62 by ATM following DSB formation. In addition to Chk2, ATM-dependent DDR involves phosphorylation of a multitude of molecules, including (but not limited to) MRE11, RAD9, RAD50, and p53 [110].

Again, ATR signaling is activated to a variety of DNA lesions compared to ATM (which is primarily activated in the presence of DSB formation). Unlike ATM, ATR cannot interact with DNA directly. Instead, ATR interacts with complexes formed by nucleofilaments between replication protein A heterotrimer and ssDNA for DNA binding [110,113]. Interestingly, DNA topoisomerase 2-binding protein (TopBP1) contains an ATR-activation domain that can mediate its activation in the absence of DNA damage [110]. Thus, this can also initiate checkpoint signaling in the absence of DNA damage. Following ATR activation, Chk1 is phosphorylated by genotoxic stress.

### 5.2. The Role of DNA Damage in PH in COPD

In COPD and PH, it is well-known that ROS generation, oxidative stress, and inflammation are increased. These cellular processes that play a key role in the development and progression of these respiratory diseases are also known to increase DNA damage. The increase in DNA damage seen in PH in COPD influences a highly proliferative and anti-apoptotic state, contributing to PA vascular remodeling [107,108,109]. High levels of DNA damage markers (i.e., γ-H2AX and 53BP1) have been reported in human PH lung samples, as well as illustrated in animal models of PH [108,109,114]. Studies have shown that cellular damage resulting from CS-induced oxidative stress is partially controlled by lack of ATM kinase activity.

It is well-accepted that DNA damage signaling leads to cell cycle arrest, decreased or inhibited proliferation, and ultimately cell death. However, in the context to PH and PASMCs, this is not the case [108]. An imbalance between proliferation and cell death suggests that DNA damage acquired from exposure to cigarette smoke or nicotine can influence the vasoremodeling seen in COPD-associated PH. Given the crucial role of RISP-mediated ROS generation in COPD PASMCs and the large role that ROS generation plays on oxidative stress-induced DNA damage, examining the specific role of RISP-mediated DNA damage on PA vasoremodeling in COPD and PH may be a likely important avenue to develop future effective therapeutics.

### 5.3. Therapeutic Effects of DNA Damage Inhibitors on PH in COPD

In studies using PASMCs, inhibition of different DDR molecules has shown to down-regulate DDR target genes (i.e., related to PARP1, EYA3, and PIM1), reduce proliferation, and increase apoptosis [109]. All these are highly involved in PA vasoremodeling and PH in COPD. Presumably, inhibiting these processes pose high therapeutic potential in treating PH in COPD. However, dysfunctional DDR or altered DNA damage may be the result of other signaling pathways, such as inflammation, hypoxia, etc. Thus, it is important to delineate the source of DNA damage to create specific DDR-targeted treatments.

## 6. Inflammation

It has been well-established that inflammation plays a significant role in the development and progression of COPD and PH. Inflammation in PH in COPD can contribute to vasoconstriction and vascular remodeling through the recruitment of inflammatory cells, such as neutrophils and macrophages [95,115]. Exposure to CS and nicotine can activate these inflammatory cells and cause a cascade of inflammatory signaling.

### 6.1. Major Inflammatory Signaling Pathways

Inflammatory signaling pathways are often triggered when innate immune cells (i.e., neutrophils and macrophages) detect infection or injury. They are the first line of defense in innate immunity and inflammation. These cells express pattern recognition receptors (PRRs) that detect microbial components, pathogen-associated molecular patterns (PAMPs), and damage-associated molecular patterns (DAMPs) [116,117]. PAMPs and DAMPs are specific molecules that are released from necrotic cells and/or damaged tissues. Different PRRs distinctly respond to PAMPs and DAMPs; however, a common event in PRRs is activation of the NF-κB inflammatory signaling pathway [116,117]. Although there are many inflammatory signaling pathways (JAK-STAT3, MAPK, PI3K, and non-classical inflammatory pathways), this section will focus on the importance and prevalence of NF-κB signaling in PH in COPD.

NF-κB is a family of inducible transcription factors involved in regulating inflammatory genes. There are five structurally-related family subunits including p50, p52, RelA or p65, RelB and c-Rel [116]. These subunits mediate target gene transcription by binding specific DNA element called the κB enhancer. The NF-κB proteins are then sequestered in the cytoplasm by inhibitory proteins, which include the IκB family members [116,118].

NF-κB activation involves two major signaling pathways: the canonical and non-canonical pathways. Both pathways are important in regulating immune and inflammatory responses [116,119]. In the canonical NF-κB pathway, stimuli such as ligands of different cytokine receptors, TNF receptors, and B-cell receptors, induce the degradation of inhibitory protein IκBα. This IκBα degradation is initiated by site-specific phosphorylation by IκB kinase (IKK). Once IKK is activated, the kinase phosphorylates IκBα at two N-terminal serine residues, which then triggers ubiquitin-dependent degradation of IκBα in the proteosome. Transient nuclear translocation of canonical NF-κB members, specifically p60/p65 and p50/c-Rel dimers, is the result of inhibitory protein IκBα degradation [116,118,119].

Non-canonical NF-κB signaling selectively responds to stimuli but does not rely on the degradation of IκBα. Instead, non-canonical signaling relies on processing p65 precursor protein, p100. Phosphorylated p100 induces ubiquitination and processing, involving the degradation of its C-terminal IκB-like structure. This generates a mature NF-κB subunit, p52, and then causes nuclear translation of non-canonical p52/RelB [116,118,119].

### 6.2. The Role of NF-κB-Mediated Inflammation in COPD and PH

Sputum and BALF specimens are used as common clinical assessments of inflammation in patients with respiratory diseases, such as COPD and also asthma. In sputum and BALF collected from COPD patients, alveolar macrophages and neutrophils were elevated [95,96,120]. This elevation of inflammatory cells may be attributed to acute or chronic exposure to CS. Importantly, activated alveolar macrophages can release pro-inflammatory mediators including tumor necrosis factor (TNF)-α, interleukin (IL)-1 and IL-6 [95]. These pro-inflammatory mediates are involved in an inflammatory response, but can also induce inflammation-driving transportation factors such as NF-κB and STAT3, which are highly upregulated in COPD [95,96].

Additionally, there are upregulated NF-κB activation and increased inflammatory cells in COPD patients, observed through bronchial biopsy [120,121]. IκBα levels are significantly lower in tissues and fluids collected from COPD patients compared to non-smoking healthy patients [97,120]. IKK activity, responsible for IκBα degradation, is also increased in COPD patients and smokers [97,120]. Neutrophils isolated from COPD sputum and BALF show increased NF-κB activation following CS exposure [120,122]. Macrophages isolated from BALF collected from patients with PH also show activated NF-κB [123].

Increased NF-κB activity has also been shown to be involved in asthmatic airway hyperresponsiveness and remodeling [28,120]. A study by Tully et al. [28] demonstrated that epithelial activation of NF-κB is required for airway remodeling and hyperresponsiveness following dust mite-induced inflammation. Inhibition of NF-κB diminishes neutrophil recruitment, airway remodeling and hyperresponsiveness in asthma, suggesting a potential targetable role in PA vasoremodeling and PH.

Studies have shown activated of NF-κB in PASMCs of the pulmonary vessel in idiopathic PH [98]; however, the role of NF-κB on remodeling and constriction has not been well-tested. Another study illustrated that inhibition of NF-κB using IMD-0354 blocked hyperproliferation of PASMCs in a monocrotaline-induced PH animal model [124]. Additionally, the role of NF-κB in a hypoxia-induced PH model in PASMCs shown NF-κB-mediated HIF-1α expression as a hypoxia-regulated transcription factor [125]. Accumulation of HIF-1α has been shown to inhibit hypoxia-induced apoptosis and, thus, contribute to PA vasoremodeling and PH [125,126]. A recent study using an e-cigarette exposure model demonstrated increased NF-κB-associated inflammatory cytokines as well as an increase in ROS-induced oxidative stress in murine lung tissue [93].

### 6.3. The Interplay of ROS and DNA Damage on Inflammation

It has been shown that ROS can activate NF-κB-mediated inflammatory signaling pathways [119,127]. In response to increased ROS, it is critical for cells to prevent further oxidative damage to maintain cell survival. The expression of NF-κB target genes, such as genes encoding antioxidant proteins, promote cell survival directly altering the levels of cellular ROS. It has also been documented that DNA damage can itself cause an inflammatory response, suggesting a role for ROS-mediated DNA damage-induced inflammation in COPD and PH. Here, we will discuss the interplay between ROS, DNA damage and inflammation.

ROS-dependent regulation of inflammatory signaling is interesting because studies have shown that ROS can both inhibit and promote inflammatory signaling. It has been shown that ROS can play a regulatory role on NF-κB signaling. Cytoplasmic thioredoxin, an endogenous small redox protein that maintains protein thiol groups, has been shown to block the degradation of I-κB [54,127,128]. It has also been shown that ROS can disturb or inhibit the ubiquitination and degradation of I-κB and subsequently inhibit the activation of NF-κB [54]. Conversely, nucleic thioredoxin has been shown to promote NF-κB activity by enhancing DNA binding abilities [127,128,129,130].

Cysteine residue 62 in the Rel homology domain (RHD) of NF-κB p50 subunit is prone to oxidation, decreasing its ability to bind DNA [127]. IKK is also very susceptible to oxidative stress, specifically S-glutathionylation of IKKβ on cysteine 179 [54]. This process subsequently inhibits the catalytic activity of the IKK subunit and NF-κB activity as well.

Studies from our laboratory have shown that RISP-mediated ROS regulation of RyR2 in PASMCs alter Ca^2+^ signaling and can lead to increased vasoremodeling and constriction of PAs after hypoxia exposure [3,4,44,76]. In our recent publication, RyR2 KO attenuated hypoxia-induced activation of NF-κB as shown through decreased p65/p50 nuclear translocation [44]. This study may suggest that RISP-mediated ROS generation can lead to indirect and direct activation of inflammation, influencing vasoremodeling and constriction seen in PH and COPD.

A major role of the immune system is to mediate or inhibit cellular complications arose from dangerous and invasive elements. This is accomplished through the recognition of PPRs of PAMPs and DAMPs. There is a strong overlap and intersection between receptors and adaptor proteins related to DAMP and PAMP recognition and often leads to a positive feedback loop [109,131,132]. This is evidently illustrated in cases where DNA damage acts as the stress factors, which can trigger the activation of inflammatory pathways leading to pro-inflammatory cytokine release. Chronic DNA damage triggers senescent cells to secrete senescence-associated secretory phenotype (SASP) factors, which can act as strong immune modulators [133]. Interestingly, it is known that inflammatory responses can also cause enhanced ROS generation at the site of inflammation leading to cell dysfunction and tissue injury. This inflammation-mediated ROS generation can cause DNA damage, which in turn can cause more inflammation. This positive feedback loop may be illustrated in COPD and PH, where exposure to a chronic irritant, such as cigarette smoke or nicotine, may cause inflammation [95,96,134], increasing ROS, influencing ROS-induced DNA damage leading to more inflammation. This interesting feedback loop may shine light on future research avenues in order to develop more efficacious therapeutics for COPD and its associated PH.

### 6.4. Therapeutic Effects of Anti-Inflammatory Drugs

As we previously discussed, the current treatments for COPD involve non-specific anti-inflammatories such as inhaled corticosteroids to control inflammation and swelling in the airway [135]. However, inhaled corticosteroids are not the first-line therapeutic against COPD and also often paired with bronchodilators. NF-κB is known to play a large role in regulating inflammation in COPD and its associated PH [115,116]. On the other hand, this molecule is ubiquitously expressed and has a multitude of intersections with other cellular processes. Apparently, targeting this pathway is not ideal in treating these pulmonary diseases. As a consequence, it is necessary to determine a specific and direct target for the NF-κB signaling pathway that contributes to the development of COPD and PH.

## 7. Conclusions

**Figure 1:**
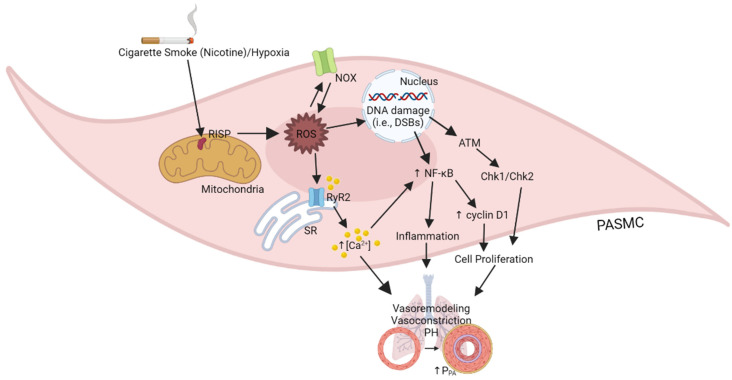
Illustration of the role of RISP-mediated ROS generation on PASM cellular responses in PH in COPD.

In this review, as illustrated in Figure 1, we have extensively described the latest important findings from a series of our studies and others. One of the specific essential findings is that the critical molecule of mitochondrial complex III, RISP, may play a potentially important role in PH in COPD by the overproduction of mitochondrial ROS in PASMCs [1,2,3,4,44]. The overproduced mitochondrial ROS subsequently induce NOX-derived cytosolic ROS generation, i.e., RIRG, by activating PKCε [57]. RISP-dependent mitochondrial ROS, together with NOX-derived cytosolic ROS, synergistically dissociate FKBP12.6 from RyR2 channels, increase the channel activity, induce SR Ca^2+^ release, leading to PA vasoconstriction, remodeling, and PH [44]. RISP-dependent increased mitochondrial ROS and NOX-derived ROS may also lead to DNA damage, ATM activation, Chk1 and Chk2 operating, and enhanced cell cycle progression, as well as inhibited apoptosis, thereby causing increased PASMC proliferation, PA vasoremodeling, and PH in COPD. Moreover, RISP-dependent, DNA damage-associated ATM signaling may activate NF-κB-mediated inflammation, contributing to PH in COPD. Moreover, drugs and biologics targeted at key molecules of these complex signaling pathways, such as RISP, ATM, and NF-κB, may become more effective and specific therapeutic options for treating PH in COPD and other devastating inflammatory lung diseases.

## Abbreviations

AHR	Airway hyperresponsiveness
ASMCs	Airway smooth muscle cells
ARE	Antioxidant response element
ATM	Ataxia-telangiectasia mutated
ATR	ATM and Rad3-related
BALF	Bronchoalveolar lavage fluid
COPD	Chronic obstructive pulmonary disease
CS	Cigarette smoke
CICR	Ca^2+^-induced Ca^2+^ release
CIRG	Ca^2+^-induced reactive oxygen species generation
TRPC	Canonical Transient Receptor Potential
DAMP	Damage-associated molecular pattern
Chk1 and Chk2	Checkpoint kinase 1 and 2
DAG	Diacylglycerol
DDR	DNA damage response
TopBP1	DNA Topoisomerase II Binding Protein 1
DSB	Double-strand break
ETC	Electron transport chain
ERK	Extracellular signal-regulated kinase
FKBP12.6	FK506 binding protein 12.6
GSH	Glutathione
GPx	Glutathione peroxidase
HPV	Hypoxic Pulmonary Vasoconstriction
IKK	IκB kinase
IP_3_	Inositol-1,4,5-triphosphate
IP_3_R	Inositol-1,4,5-triphosphate receptor
IL	Interleukin
JAK	Janus Kinase
Keap1	Kelch-like ECH-Associating protein 1
LTVDCC	L-type voltage-dependent Ca^2+^ channel
MMP	Matrix metalloprotease
MAPK	Mitogen-activated protein kinase
nAChR	Nicotinic acetylcholine receptor
NAPDH	Nicotinamide dinucleotide phosphate
NOX	NAPDH oxidase
NOS	Nitric oxide synthase
NF-κB	Nuclear factor-kappa B
Nrf	Nuclear respiratory factor
PAMP	Pathogen-associated molecular pattern
PLC	Phospholipase C
PKC	Protein kinase C
PH	Pulmonary hypertension
PA	Pulmonary artery
P_PA_	Pulmonary arterial pressure
PVR	Pulmonary vascular resistance
PDTC	Pyrrolidine dithiocarbamate
RNS	Reactive nitrogen species
ROS	Reactive oxygen species
RHD	Rel homology domain
RICR	ROS-induced Ca^2+^ release
RIRG	ROS-induced ROS generation
RISP	Rieske iron–sulfur protein
RyR	Ryanodine receptor
SR	Sarcoplasmic Reticulum
SSB	Single-strand break
SMCs	Smooth muscle cells
SOD	Superoxide Dismutase
TNF-β	Tumor necrosis factor-β
TRP	Transient receptor potential
K_V_	Voltage-dependent potassium
